# The value of blood-based measures of liver function and urate in lung cancer risk prediction: A cohort study and health economic analysis

**DOI:** 10.1016/j.canep.2023.102354

**Published:** 2023-06

**Authors:** Laura J. Horsfall, Caroline S. Clarke, Irwin Nazareth, Gareth Ambler

**Affiliations:** a Department of Primary Care and Population Health, University College London, UK; b Department of Statistical Science, University College London, UK

**Keywords:** Screening, Lung cancer, Early detection, Low-dose computed tomography, Epidemiology, Health economic evaluation, UK Biobank

## Abstract

**Background:**

Several studies have reported associations between low-cost blood-based measurements and lung cancer but their role in risk prediction is unclear. We examined the value of expanding lung cancer risk models for targeting low-dose computed tomography (LDCT), including blood measurements of liver function and urate.

**Methods:**

We analysed a cohort of 388,199 UK Biobank participants with 1873 events and calculated the c-index and fraction of new information (FNI) for models expanded to include combinations of blood measurements, lung function (forced expiratory volume in 1 s - FEV_1_), alcohol status and waist circumference. We calculated the hypothetical cost per lung cancer case detected by LDCT for different scenarios using a threshold of ≥ 1.51 % risk at 6 years.

**Results:**

The c-index was 0.805 (95 %CI:0.794–0.816) for the model containing conventional predictors. Expanding to include blood measurements increased the c-index to 0.815 (95 %CI: 0.804–0.826;p < 0.0001;FNI:0.06). Expanding to include FEV_1_, alcohol status, and waist circumference increased the c-index to 0.811 (95 %CI: 0.800–0.822;p < 0.0001;FNI: 0.04). The c-index for the fully expanded model containing all variables was 0.819 (95 %CI:0.808–0.830;p < 0.0001;FNI:0.09). Model expansion had a greater impact on the c-index and FNI for people with a history of smoking cigarettes relative to the full cohort. Compared with the conventional risk model, the expanded models reduced the number of participants meeting the criteria for LDCT screening by 15–21 %, and lung cancer cases detected by 7–8 %. The additional cost per lung cancer case detected relative to the conventional model was £ 1018 for adding blood tests and £ 9775 for the fully expanded model.

**Conclusion:**

Blood measurements of liver function and urate made a modest improvement to lung cancer risk prediction compared with a model containing conventional risk factors. There was no evidence that model expansion would improve the cost per lung cancer case detected in UK healthcare settings.

## Highlights

• Low-cost blood tests for liver function and urate enhance conventional lung cancer risk models by about 6%-10% in smokers.
• For comparison, this improvement was similar to including measures of lung function.
• Fewer people met the risk threshold for low-dose CT screening being trialled in England after blood measurements were added to the risk model.
• There was no clear health-economic benefit of adding liver function and urate testing to lung cancer screening programmes.

## 1 Introduction

Lung cancer is the most common cause of cancer mortality for men and women in the United Kingdom (UK), accounting for 21 % of all cancer deaths [1]. Unlike most cancers, survival for lung cancer has shown only minor improvements since the 1970s [1]. In the past decade, two pivotal randomised controlled trials of chest screening by low-dose computed tomography (LDCT) were completed in the United States (US) and Europe. Both trials demonstrated a long-term (6–10 years) survival improvement of 20–33 % [2], [3]. In response, the US Preventive Services Task Force (USPSTF) issued guidelines recommending annual LDCT screening for people meeting specified age and cigarette smoking history thresholds [4]. A large UK-based trial of LDCT screening (SUMMIT: NCT03934866) is ongoing but similar reductions in mortality have been reported for a smaller trial [5]. In 2019, the National Health Service (NHS) in England began introducing “Targeted Lung Health Checks” in areas with high lung cancer mortality [6], [7]. The NHS England protocol specified that individuals aged 55–74 years be referred for LDCT screening if their lung cancer risk exceeds 1.51 % by the PLCO_m2012_ model (six year risk) or 2.5 % by the Liverpool Lung Project version 2 (LLPv2) model (five year risk) [8]. Risk scores are supported by several analyses reporting improved cancer detection and cost-effectiveness over simple age and smoking thresholds currently used in the US [9], [10], [11]. Lung cancer models validated in the UK rely on self-reported smoking status and do not currently include objective measures of respiratory health or blood-based measurements. However, the SUMMIT trial and the Targeted Lung Health Checks programme are examining the value of lung function testing to measure forced expiratory volume in 1 s (FEV_1_) for improving lung cancer risk stratification [12].

Liver blood tests (LBTs) are among the most common assays requested by primary health care providers in the UK. Strong independent associations between LBT components and lung cancer have been reported in large cohort studies [13], [14], [15]. One of the blood measurements, serum bilirubin, was identified in metabolomic profiling as the only relevant biomarker for lung cancer [15]. Although less frequently ordered by primary health care providers, urate measurements are also strongly associated with lung cancer amongst people who smoke [16]. In laboratory studies, bilirubin and urate demonstrate strong antioxidant properties, suggesting these molecules may help protect respiratory tissues against oxidative stress from environmental stressors such as cigarette smoke [17], [18], [19], [20], [21], [22]. Recent genetic studies using Mendelian randomisation support a causal relationship for bilirubin but not for urate [13], [23], [24]. For other components of LBTs including the liver enzymes, the evidence of a biologically plausible or causal relationship is weaker. The associations reported in cohort studies may reflect unmeasured/misclassified causal proxies (i.e., residual confounding) or reverse causation from the undiagnosed disease process such as undetected bone metastases [14]. However, these blood measurements may still improve existing risk scores irrespective of the causal or non-causal nature of the relationships [25]. Whether these low-cost blood measurements can be repurposed to improve lung cancer risk prediction has not been evaluated.

Using a large cohort of UK residents (UK Biobank), we explored the clinical and economic value of LBTs (bilirubin, albumin, alanine aminotransferase, aspartate aminotransferase, alkaline phosphatase, gamma-glutamyl transferase) and urate in lung cancer risk prediction. We compared this to including lung function testing (forced expiratory volume in 1 s - FEV_1_), alcohol status, and waist circumference. We examined the relative improvement in prediction models and estimated the short-term cost per case detected for screening scenarios requiring different healthcare resources.

## 2 Material and methods

### 2.1 Data source

The UK Biobank resource (UKBB) is a prospective cohort study of over 500,000 UK residents recruited during 2006–2010 from age 40 years [26]. Blood samples were collected at baseline from all participants and included the blood tests evaluated in the present study (Table S1). The primary outcome is incident lung cancer recorded following study recruitment. Participants with a prior lung cancer diagnosis or reported receiving treatment for lung cancer when they attended the research centre were excluded. Cancer diagnoses in UKBB were provided by the Medical Research Information Service, based at the National Health Service Information Centre (http://www.ic.nhs.uk/services/medical-research-informationservice) for participants residing in England and Wales. The Information Services Division of NHS Scotland (http://www.isdscotland.org/HealthTopics/Cancer) provides the cancer data records for participants living in Scotland. These national cancer registries obtain information from various sources, including hospitals, treatment centres, hospices and nursing homes, private hospitals, general practices, death certificates, and Hospital Episode Statistics. NHS Digital provides data on UK Biobank participants who have died in England & Wales. The NHS Central Register (NHSCR), part of the National Records of Scotland, provides data for participants in Scotland. Diagnoses and causes of death are coded using the International Classification of Disease (ICD) versions 9 and 10. We defined our outcome as malignant neoplasms of the trachea and bronchus (ICD10: C33–C34). A self-reported cancer diagnosis is also available and used in addition to ICD codes to identify participants’ cancer history. Participants entered the cohort when they attended the UKBB regional research centres. They were censored at the earliest of the following dates: lung cancer diagnosis, loss to follow-up due to emigration, death, or end of the follow-up period. The most recent date of complete monitoring for incident cancers at the time of analysis was March 31st, 2016, for England and Wales and October 31st, 2015, for Scotland. We excluded people who no longer wished to participate (n = 5167) and those with a history of lung cancer at recruitment (n = 527). This research has been conducted using the UK Biobank Resource under Application no. 5167. UK Biobank received ethics approval from the National Health Service National Research Ethics Service (Ref: 11/NW/0382).

### 2.2 Statistical analysis

We fitted flexible Royston-Parmar (R-P) proportional hazards models with time since attending the UKBB recruitment centre as the timescale [27], [28]. R-P models use restricted cubic splines to capture the functional form of the baseline hazard. We investigated four risk model scenarios based on expected resource use in the primary care setting. For the conventional model (Scenario 1), we added variables used in LLPv2 and PLCO_m2012_ lung cancer risk tools. These included age, sex, ethnicity, timescale, smoking status, pack-years of cigarette smoking, family history of lung cancer, social deprivation measured by Townsend score [29], history of cancer, asthma, allergy, tuberculosis, pneumonia, emphysema, asbestos exposure. The quality and completeness of these conventional variables in the primary care record are low in the UK. A phone call or questionnaire would be required to implement screening Scenario 1 [30]. For Scenario 2, we added to Scenario 1 measurements of FEV_1_ and other variables that may help identify high-risk participants without requiring tests or procedures (alcohol intake and waist circumference) [31], [32]. Obtaining these variables would require an appointment with a health professional trained in spirometry. In the third scenario, we added LBTs and urate to the conventional model in Scenario 1. In terms of health service resource use, obtaining these variables would require the patient to attend a blood test appointment. In the UK, LBTs are typically ordered as a battery of assays without the possibility of selecting individual tests. The final scenario (Scenario 4) included all variables mentioned above and would require the patient to attend a spirometry appointment and provide a blood sample.

We used the Akaike Information Criterion (AIC) to select the functional forms of continuous variables (linear, log-transformed, or restricted cubic spline transformation) and interactions with conventional predictors (Supplementary materials). We calculated Harrell’s c-index and Harrell’s fraction of new information (FNI) to compare the incremental values of the expanded models [33], [34]. The FNI was calculated as one minus the ratio of χ² value for Scenario 1 to the χ² value for the alternative scenario [34]. We also performed a series of sensitivity analyses. We investigated the impact of using the Bayesian Information Criterion (BIC) instead of AIC to select the functional forms of continuous variables and interactions. We used a split-sample approach for model selection instead of the full sample. Finally, we used multivariate normal regression to impute missing continuous data and recalculated the c-indexes. Further details on these analyses are reported in the Supplementary materials.

### 2.3 Health economics

We estimated the hypothetical costs and case detection rates of the four screening scenarios applied to UKBB participants with a history of smoking cigarettes (n = 106,738). The economic outcome was cost per lung cancer case detected following a single screen. We included participants if they met the criteria used for Targeted Lung Health Checks in England [7]. These criteria were ever smokers aged between 55 and 74 and with an estimated 6 year lung cancer risk of ≥ 1.51 % according to our conventional model (Scenario 1). We then estimated the additional cost per lung cancer case detected of adding follow-up blood tests and/or spirometry to re-calculate risk from the perspective of the health service provider. Based on data from the completed European lung cancer screening trial, we estimated that a single CT scan of high-risk people would correctly identify 20 % of cases at first screen [3]. Cost data were taken from published sources and inflated to 2022 prices using a web-based tool developed as a joint initiative between The Campbell and Cochrane Economics Methods Group (CCEMG) and the Evidence for Policy and Practice Information and Coordinating Centre (EPPI-Centre) [35]. We carried out one-way sensitivity analyses to examine the impact of model selection criteria, social deprivation and altering the threshold for a referral from ≥ 1.51 %. The additional tests may also help identify other underlying health issues and we calculated the proportion of participants with evidence of obstructive lung disease or alcoholic liver disease based on the results of follow-up blood/respiratory tests. Further details on the economic modelling are in the Supplementary materials.

Statistical analyses were completed using Stata version 16.1 and R version 4.0.5.

## 3 Results

The total number of participants was 501,839, with 2643 lung cancer cases during the follow-up period (Table 1). The median follow-up was seven years for the full cohort and the median time to lung cancer diagnosis was four years with 25 % of diagnoses made within two years (Table 1). After excluding missing data, 412,862 participants (2116 events) were available to identify the blood measurements' functional forms and 439,863 (2050) for FEV_1_. The unadjusted rates showed stronger associations for participants with a smoking history (Fig. S1). Low albumin, bilirubin, and raised liver enzymes/urate were associated with a raised risk of lung cancer for women with a smoking history. Low urate, bilirubin and albumin were associated with a raised risk of lung cancer for men with a smoking history. The associations with liver enzymes in men were inconsistent. Using the AIC for selection, we identified several interactions between blood measurements and other important predictors such as sex and smoking status (Table 2, Fig. S2). There were also time-dependent associations (i.e., non-proportional hazards) for certain blood measurements (Table 2). Overall, the direction of the association of the blood measurements with lung cancer incidence in the mutually adjusted models incorporating non-linearities, non-proportional hazards and interactions were the same as the unadjusted associations (Fig. S2).

**Table 1:** Cohort characteristics at baseline by lung cancer status during the follow-up period.

	Total	No lung cancer diagnosis	Lung cancer diagnosis	Missing data (%)
	N = 501,839	N = 499,196	N = 2643	
**Male sex**	228,688 (45.6 %)	227,254 (45.5 %)	1434 (54.3 %)	
**Median age at cohort entry (IQR)**	58.3 (50.6–63.7)	58.3 (50.5–63.7)	63.5 (59.3–66.9)	
**Median follow-up time in years (IQR)**	7.0 (6.4–7.7)	7.0 (6.4–7.7)	4.1 (2.3–5.8)	
**Mean weight in kilograms (SD)**	78.0 (15.5)	78.0 (15.5)	77.8 (15.5)	2766 (0.6)
**Mean height in centimetres (SD)**	168.5 (9.2)	168.5 (9.2)	168.4 (9.1)	2467 (0.5)
**Mean waist circumference in centimetres (SD)**	90.3 (13.5)	90.3 (13.5)	93.2 (13.4)	2155 (0.4)
**Smoking status**				
** Never**	273,374 (54.5 %)	273,009 (54.7 %)	365 (13.8 %)	
** Former**	172,624 (34.4 %)	171,441 (34.3 %)	1183 (44.8 %)	
** Current**	52,898 (10.5 %)	51,827 (10.4 %)	1071 (40.5 %)	
** Missing**	2943 (0.6 %)	2919 (0.6 %)	24 (0.9 %)	
**Alcohol drinker status**				
** Never**	22,358 (4.5 %)	22,275 (4.5 %)	83 (3.1 %)	
** Previous**	18,042 (3.6 %)	17,839 (3.6 %)	203 (7.7 %)	
** Current**	459,788 (91.6 %)	457,440 (91.6 %)	2348 (88.8 %)	
** Missing**	1651 (0.3 %)	1642 (0.3 %)	9 (0.3 %)	
Median pack years of smoking (IQR)*	19.0 (10.0–32.0)	19.0 (9.8–31.9)	37.0 (23.5–50.0)	
**Median Townsend deprivation index (IQR)**	-2.1 (− 3.6 to 0.5)	-2.1 (− 3.6 to 0.5)	-0.6 (− 3.0 to 2.7)	622 (0.1)
**Median alkaline phosphatase in U/L (IQR)**	80.4 (67.2–95.9)	80.3 (67.2–95.8)	88.9 (75.0–106.1)	32,835 (6.5)
**Median alanine aminotransferase in U/L (IQR)**	20.1 (15.4–27.4)	20.1 (15.4–27.4)	19.9 (15.3–26.3)	33,036 (6.6)
**Median aspartate aminotransferase in U/L (IQR)**	24.4 (21.0–28.8)	24.4 (21.0–28.8)	24.2 (20.7–28.9)	34,641 (6.9)
**Median gamma glutamyl transferase in U/L (IQR)**	26.3 (18.5–40.9)	26.3 (18.5–40.9)	31.0 (21.7–49.4)	33,092 (6.6)
**Median total bilirubin in μmol/L (IQR)**	8.1 (6.4–10.4)	8.1 (6.4–10.4)	7.6 (6.1–9.7)	34,876 (6.9)
**Median urate in μmol/L (IQR)**	302.9 (250.4–360.8)	302.9 (250.4–360.7)	313.6 (258.5–375.0)	33,410 (6.7)
**Median albumin in g/L (IQR)**	45.2 (43.5–46.9)	45.2 (43.5–46.9)	44.5 (42.7–46.2)	72,329 (14.4)
**Lung cancer in first degree relative**	62,079 (12.4 %)	61,503 (12.3 %)	576 (21.8 %)	
**History of emphysema**	12,045 (2.4 %)	11,718 (2.3 %)	327 (12.4 %)	
**History of asthma**	62,437 (12.4 %)	62,078 (12.4%)	359 (13.6 %)	
**History of allergy and/or eczema**	121,902 (24.3 %)	121,435 (24.3 %)	467 (17.7 %)	
**History of tuberculosis**	2535 (0.5 %)	2499 (0.5 %)	36 (1.4 %)	
**History of pneumonia**	6885 (1.4 %)	6829 (1.4 %)	56 (2.1 %)	
**History of cancer**	52,916 (10.5 %)	52,414 (10.5 %)	502 (19.0 %)	
**Ethnic identity**				
** White**	472,060 (94.1 %)	469,497 (94.1%)	2563 (97.0 %)	
** Asian**	11,448 (2.3 %)	11,427 (2.3 %)	21 (0.8 %)	
** Black**	8053 (1.6 %)	8036 (1.6 %)	17 (0.6 %)	
** Mixed**	2952 (0.6 %)	2937 (0.6 %)	15 (0.6 %)	
** Other**	4553 (0.9 %)	4540 (0.9 %)	13 (0.5 %)	
** Missing**	2773 (0.6 %)	2759 (0.6 %)	14 (0.5 %)	
**Mean FEV_1 in litres (SD)_**	2.8 (0.8)	2.8 (0.8)	2.4 (0.7)	48,492 (9.7)

FEV_1_ = forced expiratory volume in 1 s; U/L = Units per litre.

* Median values (interquartile range).

**Pack years of smoking were calculated for 150,539 participants who reported regularly smoking at least one cigarette per day.

**Table 2:** Lung cancer risk model specification expanded to include additional continuous variables with transformations and interactions selected using the Akaike Information Criterion.

		Transformation*		Interaction term selected			
		First transformation	Spline transformation	Sex	Age	Smoking status	Timescale (i.e., non-proportional hazards)
**Liver blood tests**	Total bilirubin	Log				Yes	2df
	Albumin	Log					
	Aspartate aminotransferase	Log	2df	Yes			2df
	Alkaline phosphatase	Log		Yes		Yes	2df
	Alanine aminotransferase	Log		Yes		Yes	
	Gamma glutamyl transferase	Log				Yes	
**Other blood tests**	Urate	Log		Yes		Yes	
**Spirometry**	FEV_1_	None (linear)	3df				
	Waist circumference	Log					

df = degrees of freedom; FEV_1_ = forced expiratory volume in 1 s.

*Knot positions for restricted cubic spline transformations are placed at Harrell’s default percentiles.

After excluding missing data, 388,199 participants with 1873 events were included in the c-index calculation for the various risk models. Scenario 2 (containing FEV_1_) increased the c-index from 0.805 in Scenario 1 (conventional predictors) to 0.811 (Table 3). Scenario 3 (containing blood measures) increased the c-index to 0.815 (Table 3). Expanding to Scenario 4 (fully expanded model) increased the c-index to 0.819 (Table 3). The fraction of new information (FNI) was 0.06 for Scenario 3 (blood test measures) and 0.04 for Scenario 2 (containing FEV_1_) (Table 3). The FNI Scenario 4 (fully expanded model) was 0.09 (Table 3). For people who met the criteria used for Targeted Lung Health Checks in England, the FNI was higher at 0.11 for Scenario 3 (containing blood measures), 0.10 for Scenario 2 (containing FEV_1_), and 0.17 for Scenario 4 (fully expanded model) (Table 4). Expanding the models resulted in a lower median predicted risk of lung cancer for Scenarios 2–4 (Table 4). The shrinkage factors suggested little optimism that needs correction due to the large sample size (Table 3). Other model selection methods had a minimal impact on the c-index but reduced the FNI (see Supplementary materials and Tables S2–S4). Multiple imputation of missing data did not materially impact the incremental changes in the c-index across the scenarios (Table S5).

**Table 3:** C-index and fraction of new information for different lung cancer risk models.

		C-index (95 %CI)	Degrees of freedom	Model likelihood ratio test (χ²)	Likelihood ratio test p-value versus Scenario 1	Fraction of new information*	Heuristic shrinkage factor**
**Scenario 1**	Basic model	0.805 (0.794–0.816)	24	3613	Ref	Ref	0.99
**Scenario 2**	Basic model + FEV_1_ + alcohol + waist circumference	0.811 (0.800–0.822)	30	3781	1.75E-33	0.04	0.99
**Scenario 3**	Basic model + liver blood tests + urate	0.815 (0.804–0.826)	70	3830	5.17E-24	0.06	0.98
**Scenario 4**	Scenario 2 + 3	0.819 (0.808–0.830)	76	3966	3.43E-46	0.09	0.98

FEV_1_ = forced expiratory volume in 1 s.

* Fraction of new information calculated as one minus the ratio of χ² value for Scenario 1 to the χ² value for the alternative scenario.

* * Shrinkage calculated as (χ² value - degrees of freedom)/χ² value

**Table 4:** Changes in predicted risk of lung cancer across different risk models applied to 106,738 UK Biobank participants with a history of ever smoking and aged 55–74 years.

		C-index (95 %CI)	Fraction of new information compared with Scenario 1*	Predicted risk of lung cancer at 2 years	Predicted risk of lung cancer at 6 years
				Median predicted risk % (IQR)	Median predicted risk % (IQR)
**Scenario 1**	Basic model	0.705 (0.691–0.720)	Ref	0.13 (0.07–0.32)	0.50 (0.28–1.26)
**Scenario 2**	Basic model + FEV_1_ + alcohol + waist circumference	0.722 (0.708–0.736)	0.10	0.12 (0.07–0.30)	0.49 (0.26–1.21)
**Scenario 3**	Basic model + liver blood tests + urate	0.725 (0.711–0.739)	0.11	0.12 (0.06–0.30)	0.49 (0.26–1.21)
**Scenario 4**	Scenario 2 + 3	0.737 (0.723–0.750)	0.17	0.12 (0.06–0.29)	0.48 (0.25–1.17)

FEV_1_ = forced expiratory volume in 1 s.

* Fraction of new information calculated as one minus the ratio of χ² value for Scenario 1 to the χ² value for the alternative scenario.

Using the same LDCT referral threshold as NHS England’s Targeted Lung Health Checks Programme (≥ 1.51 % at 6 years), we found that the expanded models reduced LDCT referrals by 15–21 % and reduced cases detected by 7–8 % (Table 5). The additional cost per lung cancer case detected was £ 8049 for Scenario 2 (containing FEV_1_) versus Scenario 3 (containing blood measures). This difference was driven by the higher cost of obtaining spirometry measures compared with standard blood tests (Table 5). Overall, Scenario 1 (conventional model) had the lowest cost per case detected at £ 25,926 (Table 5). This remained the case across all sensitivity analyses (Tables S6-S10). The overall cost per case detected across the screening scenarios was sensitive to levels of social deprivation (Table S9). For example, the cost per lung cancer case detected for Scenario 1 (conventional model) applied to people living in the least socially deprived quintile category was £ 48,527 compared with £ 19,883 in the most socially deprived quintile category (Table S9). Of the participants with an estimated 6 year lung cancer risk of ≥ 1.51 % predicted using Scenario 1 (conventional model), 4.5 % had an aspartate aminotransferase to alanine aminotransferase ratio > 2, suggesting liver damage. In contrast, 35 % of participants without a lung disease diagnosis at cohort entry had a prebronchodilator FEV_1_/FVC ratio < 0.7, suggesting obstructive lung disease.

**Table 5:** Health economic model of hypothetical lung cancer screening scenarios applied to 106,738 UK Biobank participants aged 55–74 years and with a history of ever smoking cigarettes.

Screening scenario		Total cost of initial screening via telephone contact (n = 106,738)* (£)	Per patient cost of follow-up tests to recalculate risk (£)	Total cost of follow-up tests after initial screening for those with a 1.51 % risk over 6 years (n = 22,109) (£)	No. meeting LDCT referral risk threshold (1.51 % over 6 years)	Total cost LDCT screens for those meeting risk threshold (£)**	No. participants meeting LDCT referral risk threshold and with a lung cancer diagnosis by 6 years (% of screens)	Estimated cases detected at first screen*** (% of screens)	Cost per case detected (£)
**1**	Basic model	2,195,601	None	None	22,109	2,195,601	819 (3.7)	164 (0.74)	25,926
**2**	Basic model + FEV_1_ + alcohol + waist circumference	2,195,601	63.42	2,195,601	18,930	3,597,753	765 (4.0)	153 (0.81)	34,993
**3**	Basic model + liver blood tests + urate	2,195,601	7.16	3,597,753	18,599	2,353,901	757 (4.1)	151 (0.81)	26,944
**4**	Scenario 2 + 3	2,195,601	70.58	2,353,901	17,468	3,756,054	753 (4.3)	151 (0.86)	35,701

LDCT = Low-dose computed tomography; FEV_1_ = forced expiratory volume in 1 s.

*Cost of an initial screen by a health professional using telephone contact is £ 20.57 [45]. See Supplementary information for further information on costs, resource use and assumptions on screening effectiveness.

**Cost of a low-dose computed tomography scan for one body region is £ 92.77 according to NHS National Schedule of Reference Costs for years 2019/20 and inflated to 2022 values [46].

***Estimated 20 % of lung cancer diagnoses are detected at the first screen based on the NELSON European trial of LDCT screening [3].

## 4 Discussion

We found that blood-based liver function and urate measurements improved lung cancer risk prediction, particularly for UKBB participants with a smoking history. The improvement in risk prediction from expanding to include blood-based measurements was similar to the expansion to include FEV_1_, alcohol and waist circumference. This improvement in lung cancer risk prediction for most participants resulted in a lower predicted risk than the conventional model. In our health economic analyses, applying these expanded models led to fewer participants meeting the current risk thresholds for lung cancer LDCT screening in England. However, this also meant fewer cases were detected. Our health economic analysis suggested that the additional costs of obtaining blood measurements were not offset by the cost savings of performing fewer LDCT scans. Based on these findings, we do not recommend translating these blood tests into clinical practice for lung cancer screening programmes.

The observed increase in the c-index of our expanded risk models was modest compared with studies of other predictors. For example, the inclusion of certain genetic variants increased the c-index from 0.75 (95 % CI 0.73–0.77) to 0.81 (95 % CI 0.79–0.83) [36]. Similar increases in the c-index were reported for a four-marker protein panel (0.85 (95 % CI 0.82–0.88)) when compared with a conventional risk model (0.80 (95 % CI 0.77–0.83)) [37]. Whether genotyping and protein profiling can improve the cost-effectiveness of lung cancer screening programmes is an interesting area for further investigation. Comparison of our findings with other expanded models for lung cancer is limited by the differences in specification of the reference models and the range of different statistics used to evaluate performance. The Net Reclassification Index is particularly popular for evaluating the improvement in the prediction performance but has been criticised along with measures of sensitivity and specificity [34], [38], [39]. We avoided these measures in the present study. Estimates for incremental cost per quality-adjusted life year (QALY) gained for UK-based lung cancer screening programmes versus no screening range from £ 8466/QALY gained to £ 40,034 /QALY gained due to parameter uncertainty and the wide range of possible scenarios [5], [40]. An economic evaluation of the Targeted Lung Health Checks with spirometry is ongoing. Our results by levels of social deprivation support the current strategy of focusing on areas with high lung cancer mortality rates.

This study's strengths are the cohort size and the use of more flexible and informative statistics to interpret the added value of new predictors. We demonstrated that strong observational associations between exposures and outcomes do not always translate into clinically useful improvements in prediction models. Our health economic analysis adds a cost perspective to expanding risk models in lung cancer screening. The cost-effectiveness of new risk models is rarely appraised despite the importance for healthcare decision-making and resource allocation. Limitations are that the reported relationships between blood test results and lung cancer cannot be interpreted causally due to mutual adjustment without consideration of the underlying mechanistic relationships. Our analysis used the results of single blood extraction from UKBB participants, and we could not account for intra-individual variation in test results. Two or more measurements could have provided more reliable results and improved the predictive value of the blood measurements. UKBB is often criticised for the lack of representativeness that can lead to selection bias. Respiratory disease and lower socioeconomic groups are particularly underrepresented relative to the general population. Although, it is worth highlighting that lung cancer is the leading cause of death in women UKBB participants and the second leading cause in men after ischemic heart disease. The extent to which selection bias is a problem for the generalisability of our results is hard to dissect, and it is plausible that the characteristics of ever smokers in UKBB could be similar to those who respond to a lung cancer screening invitation. For example, factors associated with non-uptake in the UKLS trial included lower socioeconomic group (OR = 0.56, p < 0.001), current smoking (OR = 0.70, p < 0.001), and higher affective risk perception (OR = 0.52, p < 0.001) [41]. Furthermore, the additional cost per case detected for the expanded risk models remained lowest for the conventional risk model, even in the most socially deprived quintile category. The UKBB sample was only recruited up to age 69. In contrast, England’s Targeted Lung Health Checks are eligible for up to age 74, which may reduce the generalisability of the health economic analysis. Unsurprisingly, we found spirometry in ever smokers detected signs of chronic obstructive pulmonary disease in 35 % of participants whereas LBTs detected signs of alcoholic liver damage in only 4.5 %. Similar results for detecting signs of chronic obstructive pulmonary disease are reported for the US [42] Canada [43], and the new Targeted Lung Health Checks in England [44]. It is therefore plausible that more comprehensive economic analyses that include the benefits of identifying and treating undiagnosed disease might favour the scenarios that include spirometry over conventional models. We only examined costs directly incurred by the health service provider, but blood testing might involve additional costs for patients if they need to attend geographically distant hospitals. There is also a time cost associated with obtaining and processing blood test results that might delay treatment initiation. We included FEV_1_ as a reference for the predictive value of the blood tests. However, we do not believe our economic analysis can be used to argue against spirometry for lung cancer screening without a full cost-utility analysis. Given the increased costs and decreased detection rates of the models including blood measurements of liver function and urate, we feel a broader analysis is unlikely to alter our conclusions on the lack of value of these measurements.

In summary, expanding lung cancer risk models to include blood measurements of liver function and urate improved prediction models as measured by the c-index and fraction of new information. However, adding these blood measurements to existing risk tools did not reduce healthcare costs in the short term or improve overall lung cancer detection rates.

## CRediT authorship contribution statement

**Laura Horsfall:** Conceptualization, Methodology, Software, Writing – original draft, Funding acquisition. **Caroline Clarke:** Investigation, Methodology, Writing – review & editing. **Gareth Ambler:** Investigation, Methodology, Software, Writing – review & editing. **Irwin Nazareth:** Supervision, Writing – review & editing.

## Funding

This research was funded in whole, or in part, by the Wellcome Trust [Grant 209207/Z/17/Z].

This research was funded in whole, or in part, by The Wellcome Trust [Grant 209207/Z/17/Z]. For the purpose of open access, the author has applied a CC BY public copyright licence to any Author Accepted Manuscript version arising from this submission.

## Conflicts of Interest

No conflicts for any authors.
