# Supplementary material for: The value of blood-based measures of liver function and urate in lung cancer risk prediction: A cohort study and health economic analysis
Source: Cancer Epidemiol. 2023 Jun;84:102354. doi: 10.1016/j.canep.2023.102354 (PMC10636591; doi:10.1016/j.canep.2023.102354)
Supplement: Supplementary file 1 — Supplementary material. [file mmc1.docx]

# Supplementary Materials

# Supplementary Methods

## Model development

Before model development, we removed biologically implausible values of continuous variables and winsorised at the 1^st^ and 99^th^ percentiles[1]. We selected the model that minimized the Akaike Information Criterion (AIC) to select the functional form of continuous variables (linear, log-transformed, or restricted cubic spline transformation). We included spline transformations with up to five degrees of freedom and knots placed at Harrell’s default percentiles[2]. Blood measures were right-skewed and log transformed before model entry and spline selection[3]. The AIC was also used to identify multiplicative interactions of blood measures and FEV_1_ with age, sex, and smoking status. We used a backwards stepwise selection of interaction terms to identify the model with the lowest AIC. We examined non-proportional hazards by entering blood measures and FEV_1_ as time-varying effects with up to five degrees of freedom. We used the full data and calculated shrinkage factors to assess overfitting. Shrinkage was calculated as (χ² value - degrees of freedom)/χ² value. To view the functional relationships between each blood test value and lung cancer, we predicted the incidence rate per 10,000 person-years for never, former, and heavy current smokers (20 or more packyears) while holding other variables at their most frequent (binary/categorical) or median (continuous) value. We created separate plots for interacting variables (Figures S1-S7).

Prediction literature often relies on discrimination measures, such as Harrell’s c-index, to compare the added value of new biomarkers. The c-index can take a value between 0 and 1 and gives the probability that, of two drawn at random, the person who survives the longest had the highest predicted survival. However, discrimination measures have been criticised for being insensitive to the value of new variables and additional descriptive statistics can provide more useful information[4, 5]. One proposed alternative is to calculate the “fraction of new information”. This measure is defined as the increase in the proportion of total predictive information for the expanded model relative to the conventional/simpler mode l[5]. We calculated the c-index and the fraction of new information to compare the incremental values of the expanded models [4, 5]. The fraction of new information is calculated as one minus the ratio of χ² value for Scenario 1 to the χ² value for the alternative scenario.

We used the Bayesian Information Criterion (BIC) as a sensitivity analysis to select functional forms and interactions applied to the full data. BIC imposes stricter penalties for complexity, making simpler models less prone to overfitting. The basic model (Scenario 1) was the same as with AIC selection, except that BIC was minimised with untransformed FEV_1_ and two degrees of freedom for the time scale. No interactions or spline transformations were identified using BIC for the expanded models. We randomly split the data 1:1 into training and validation sets and recalculated Harrell’s c-index with model selection using AIC. The model specification was the same as with AIC selection using the full data, except that FEV_1_ was untransformed, interactions with smoking status were excluded, and two degrees of freedom were selected for the time scale. Although data splitting is inefficient, based on earlier literature and biological plausibility, we anticipated a complex model specification with nonlinearities, interactions, and non-proportional hazards. Therefore, the automated model specification (e.g., cross-validation or bootstrapping) was not practical.

Using BIC for model selection specified a simpler model without interactions or spline transformations and the shrinkage factors were identical to two decimal places across all scenarios (Table S2 and S3). The c-index values were similar for the AIC and BIC models but the FNI for the blood test values in Scenario 2 was lower at 0.03 versus 0.06 (Table S2 and S3). The relative difference in c-index’ across the various scenarios from a split sample approach were similar (Table S4).

## Multiple Imputation

While initial model specification was done using the full data available for the variable of interest, for comparisons across models, we restricted the data to participants with complete data on all blood test values, FEV_1_ and other variables used in the score. As a sensitivity analysis, we recalculated the c-index following multiple imputation. We used multivariate normal regression to impute missing continuous data (Stata command mi impute mvn). This command uses an iterative Markov chain Monte Carlo method to impute missing values, and we used n = 10 imputations. Due to computational issues with the complex model selected by the AIC, we only present the results for the simple model selected using the BIC. We included the baseline hazard in the imputation model, the outcome (lung cancer) and all variables included in the fully expanded prediction model (Scenario 5) in the imputation equations. Imputations were successful for all but two participants. This increased the analytic sample for calculating the c-index and fraction of additional information from 388,199 participants with 1,873 events to 501,839 with 2,643 events. The relative difference in c-index’ across the various scenarios following multiple imputation were similar to the complete case analysis (Table S5).

## Health economic modelling

We applied the different screening scenarios detailed in the Methods section of the main manuscript to UK Biobank participants. The economic outcome was the additional cost per extra lung cancer case detected following a single screen. Costs and screening effectiveness data were taken from published sources and inflated to 2022 values using a web-based tool developed as a joint initiative between The Campbell and Cochrane Economics Methods Group (CCEMG) and the Evidence for Policy and Practice Information and Coordinating Centre (EPPI-Centre) (Table S6). The costs of additional investigations included staff time, and spirometry also included staff training. Further investigations of all ever smokers registered with a general practice are unlikely to be practical or cost-effective for the National Health Service. Instead, we modelled these tests as follow-up investigations for those identified as high-risk (≥ 1.51% chance of having lung cancer over 6 years) using the conventional model. We assumed applying all models would require initial case finding with patient contact by phone or questionnaire due to missing data in the patient record. We assumed the characteristics of people who attend further investigation/CT scans in a lung cancer screening programme would be similar to people with a history of smoking cigarettes who agreed to participate in UK Biobank. We did not attempt to model non-attendance. Based on data from the completed European trial of lung cancer screening, we estimated that a single CT scan of high-risk people would correctly identify 20% of cases at first screen [6]. We did not model harms associated with the investigation and treatment of findings that were eventually determined not to be lung cancer as these are reported to be low [7]**.**

## Supplementary figures

### Figure S1-a: Serum urate and unadjusted lung cancer incidence


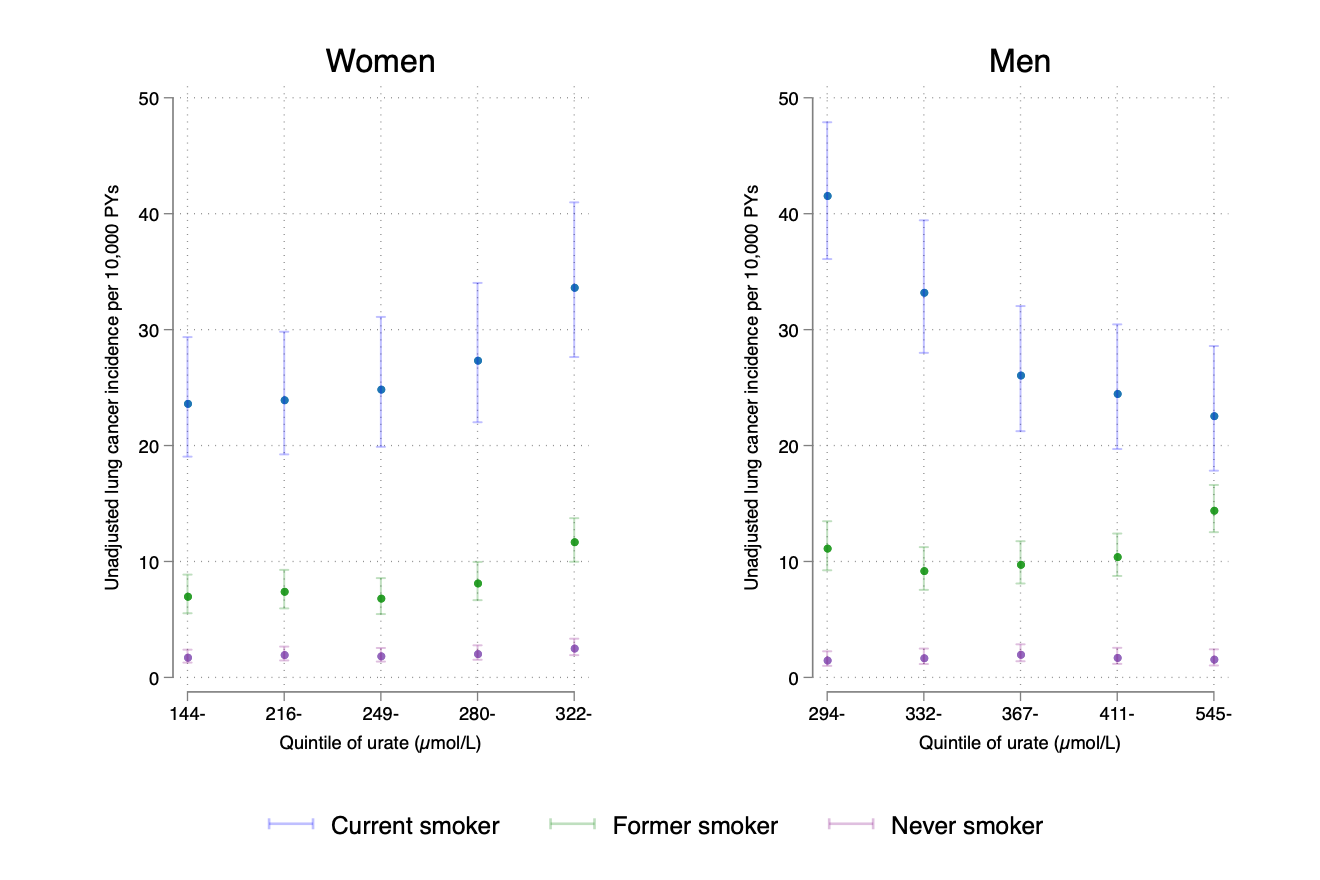


### Figure S1-b: Serum total bilirubin and unadjusted lung cancer incidence


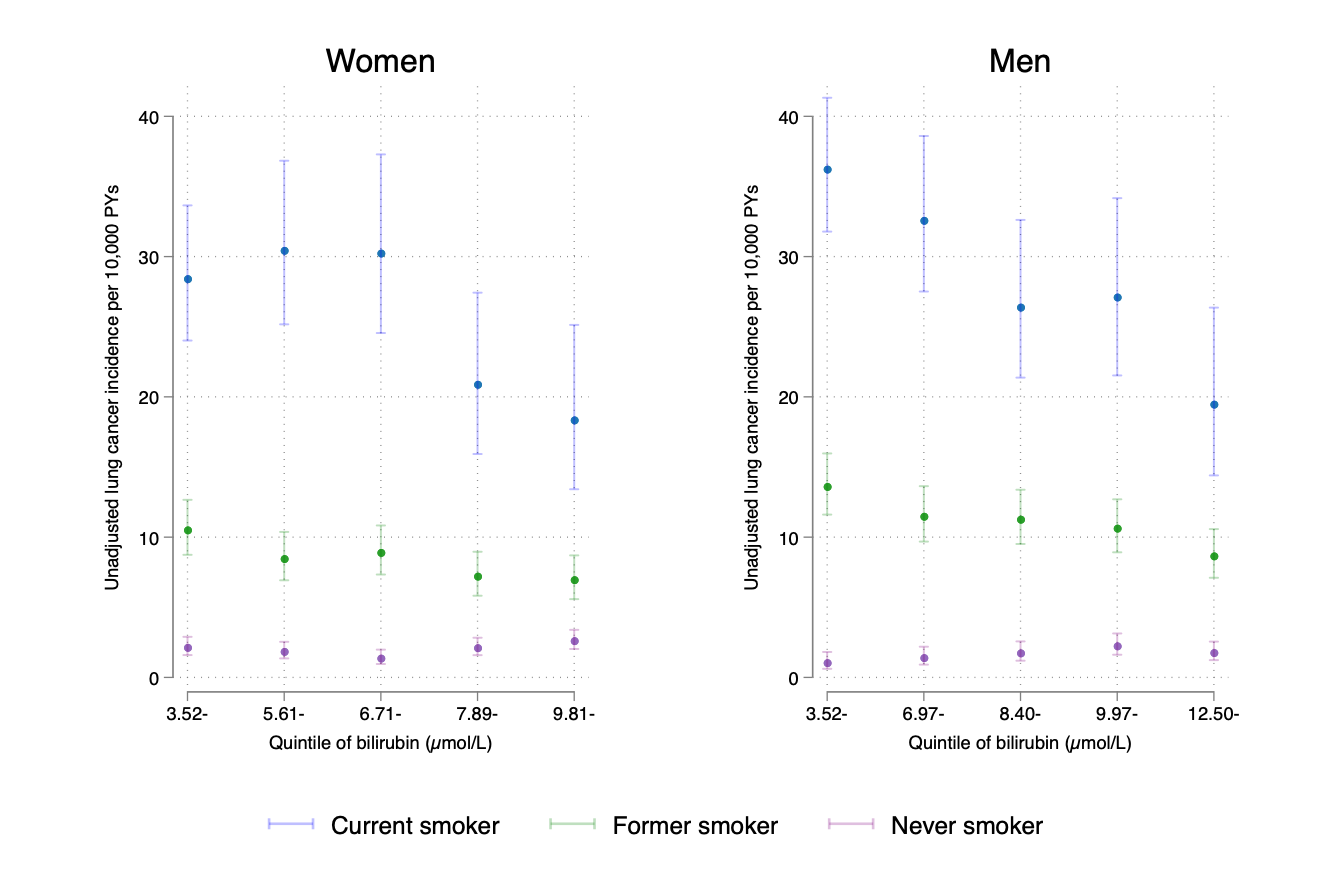


### Figure S1-c: Gamma-glutamyl transferase and unadjusted lung cancer incidence


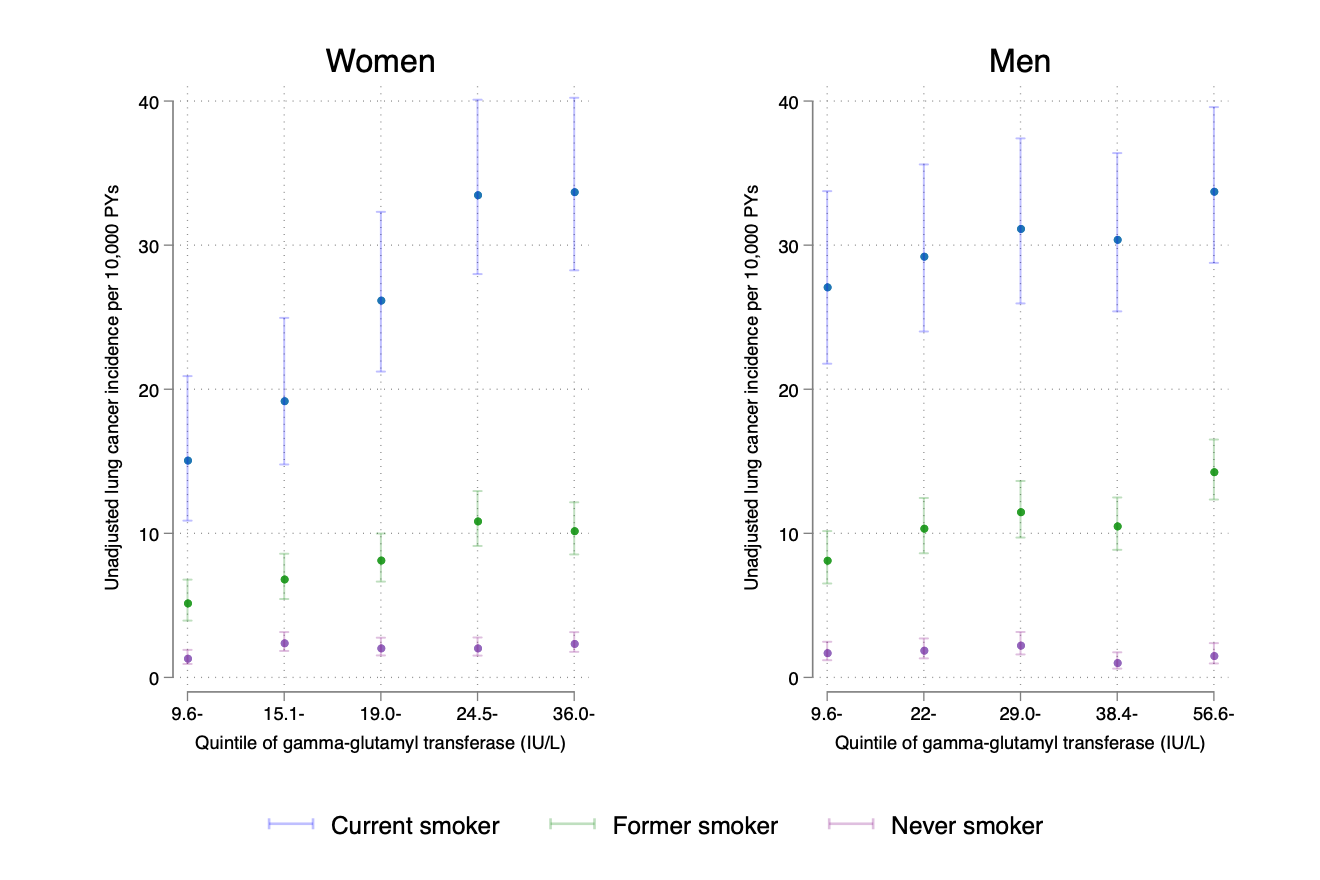


### Figure S1-d: Alkaline phosphatase and unadjusted lung cancer incidence


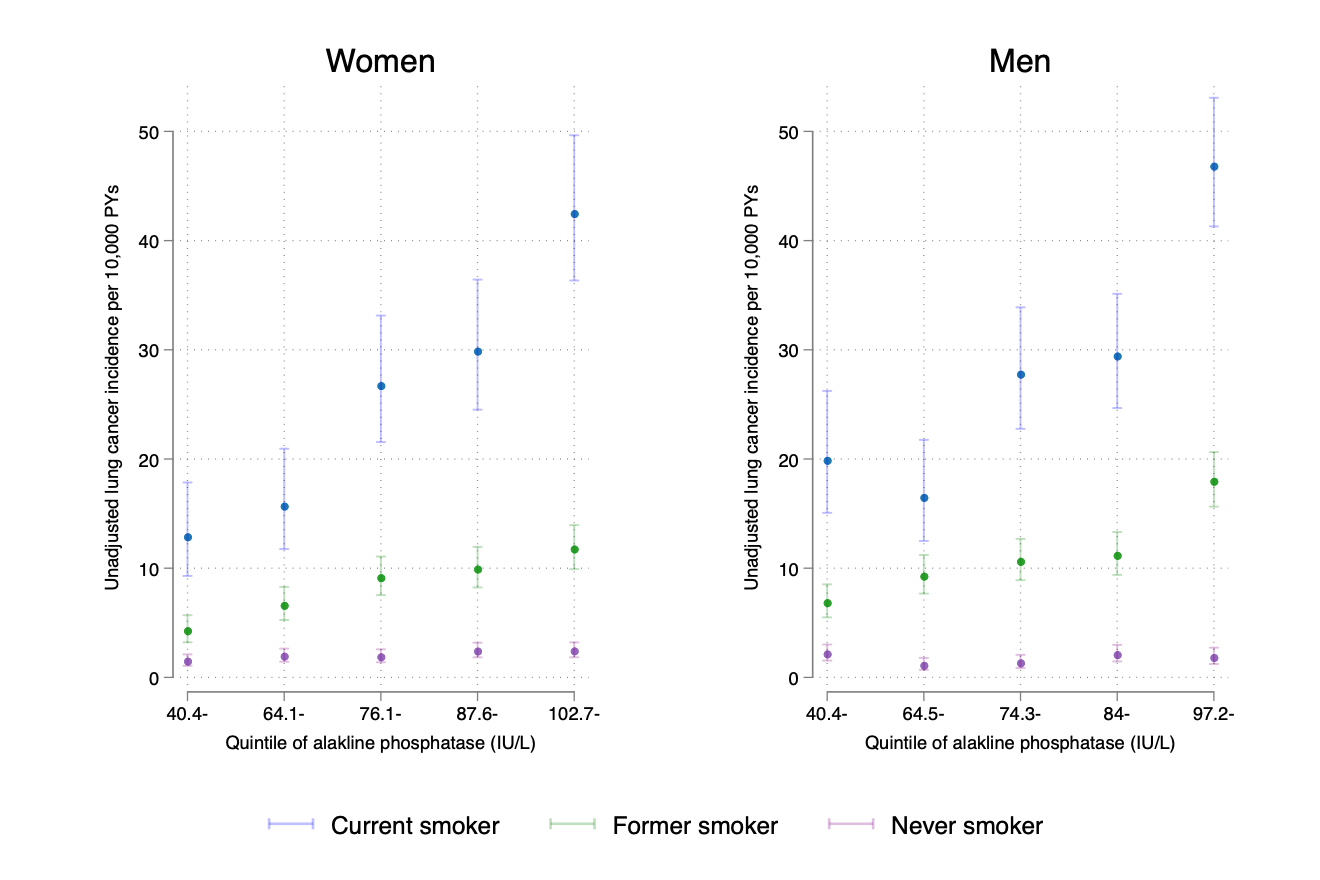


### Figure S1-e: Aspartate aminotransferase and unadjusted lung cancer incidence


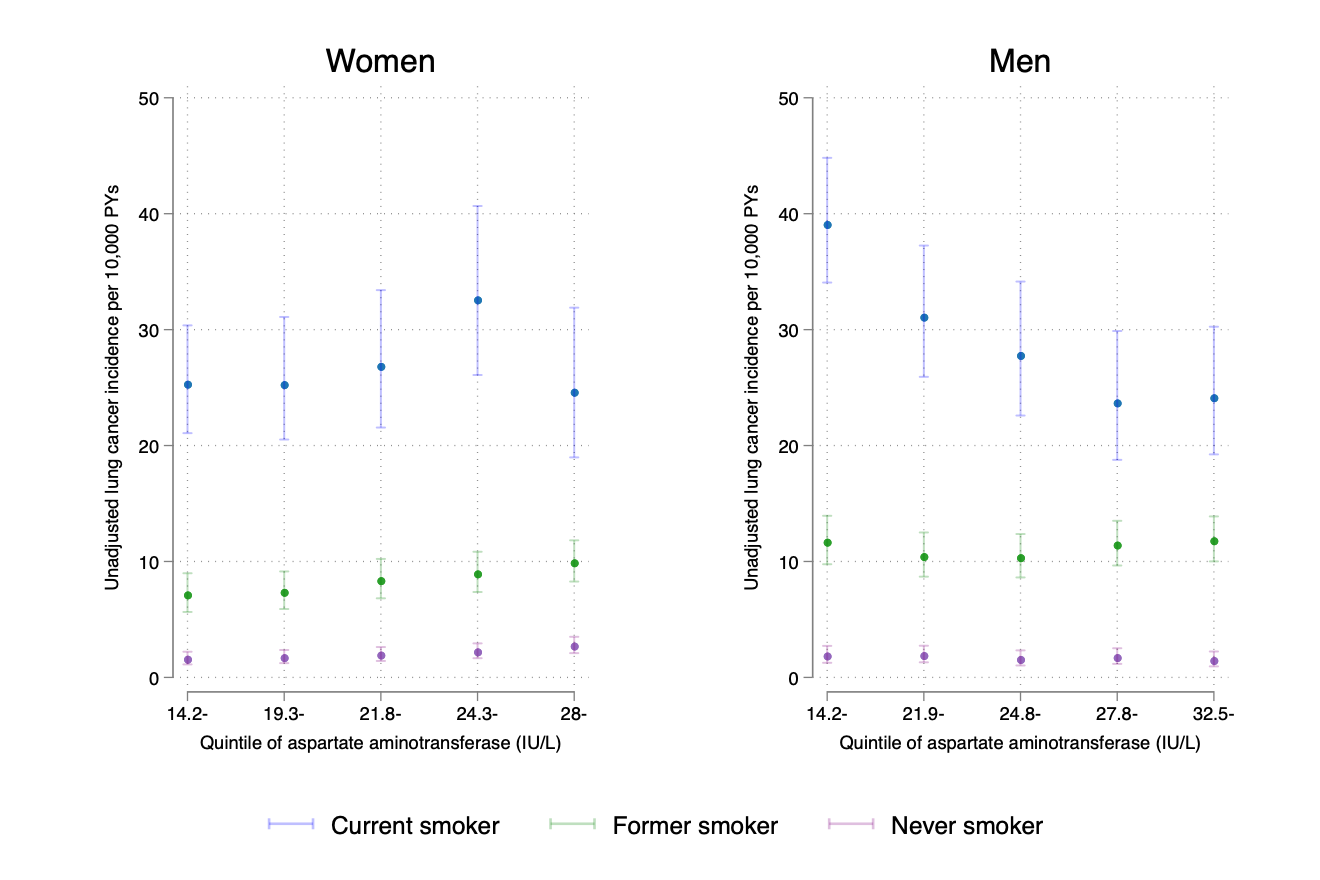


### Figure S1-f: Alanine aminotransferase and unadjusted lung cancer incidence


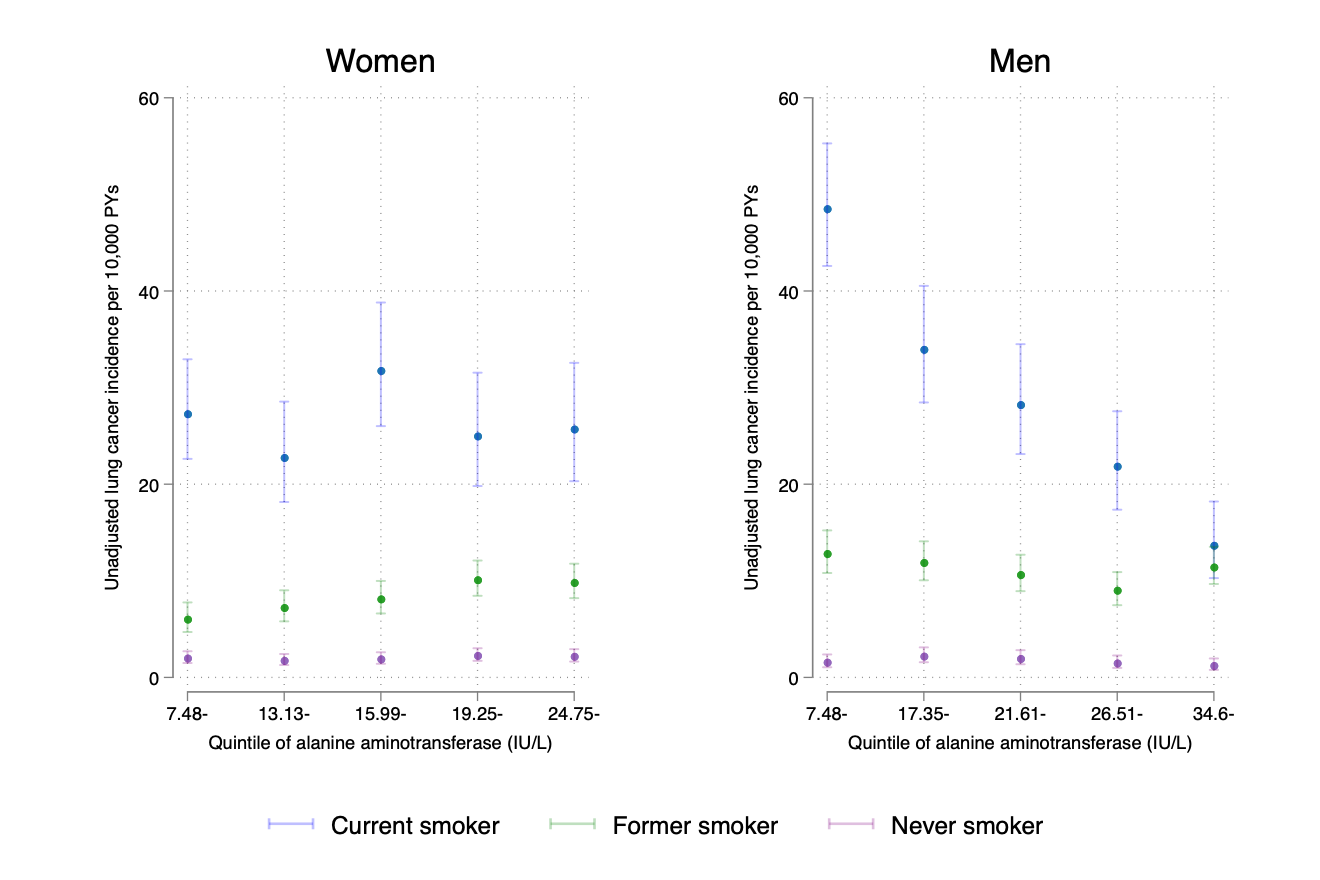


### Figure S1-g: Albumin and unadjusted lung cancer incidence


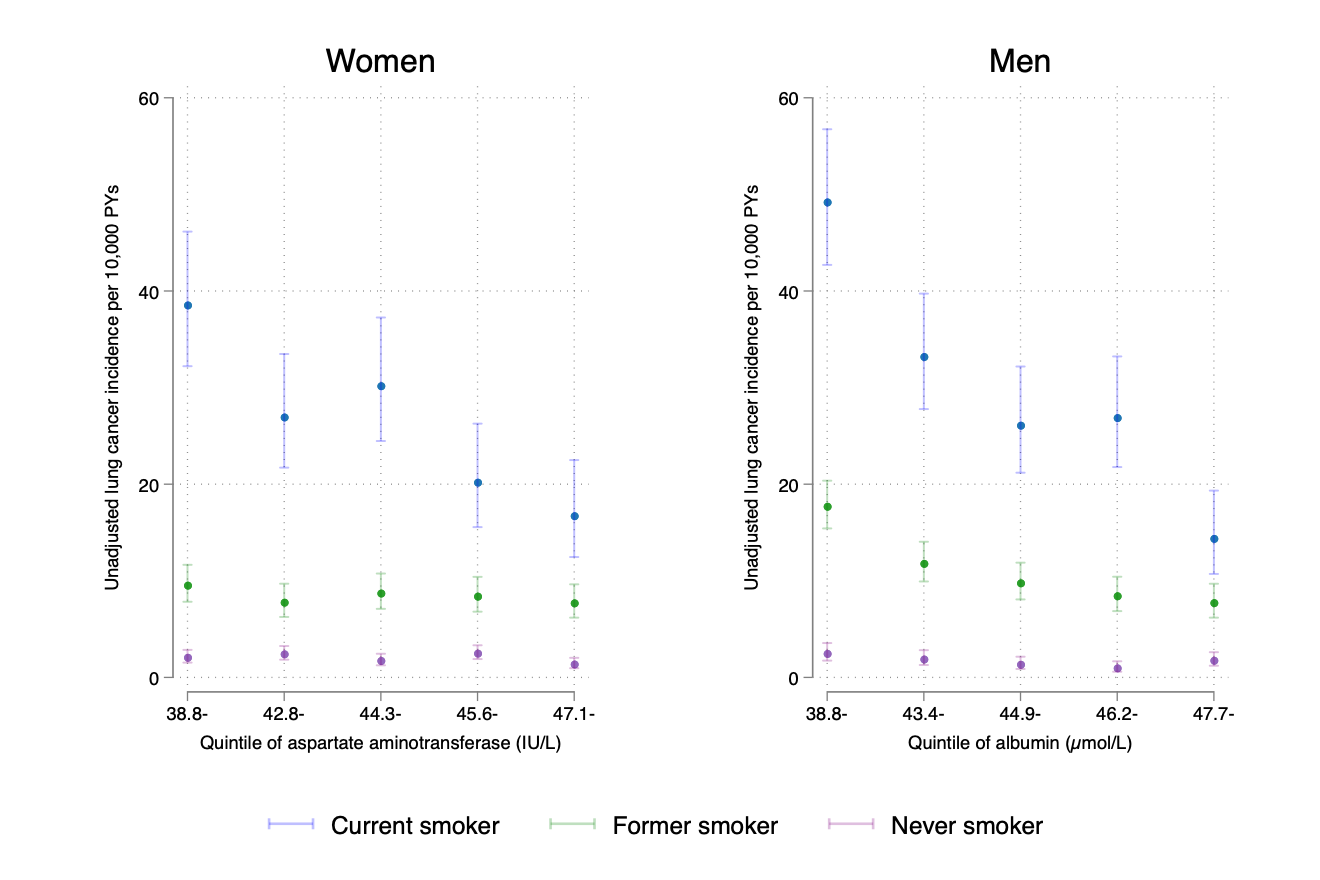


### Figure S2-a: Serum urate and predicted lung cancer incidence


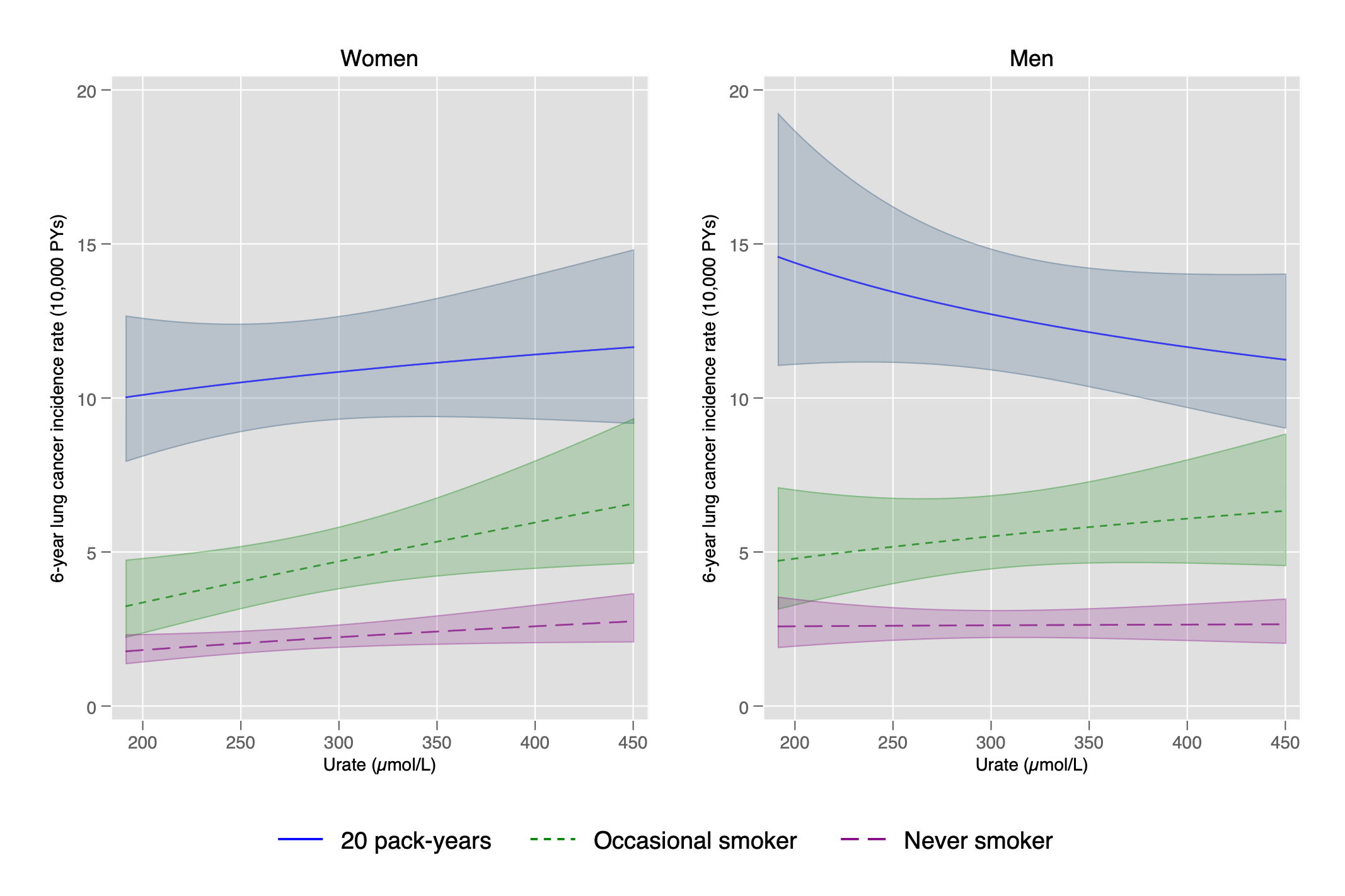


### Figure S2-b: Serum total bilirubin and predicted lung cancer incidence


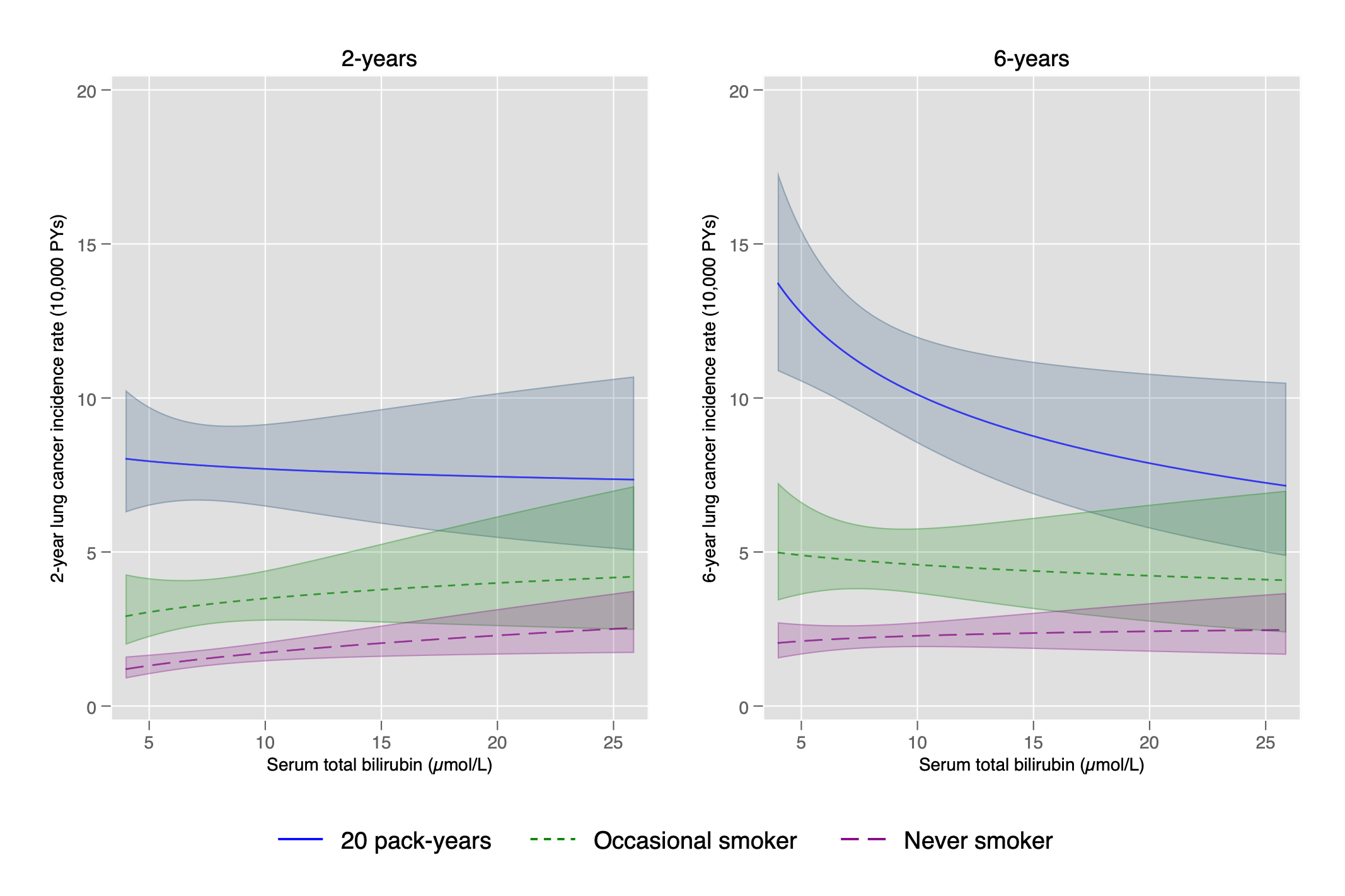


### Figure S2-c: Gamma-glutamyl transferase and predicted lung cancer incidence


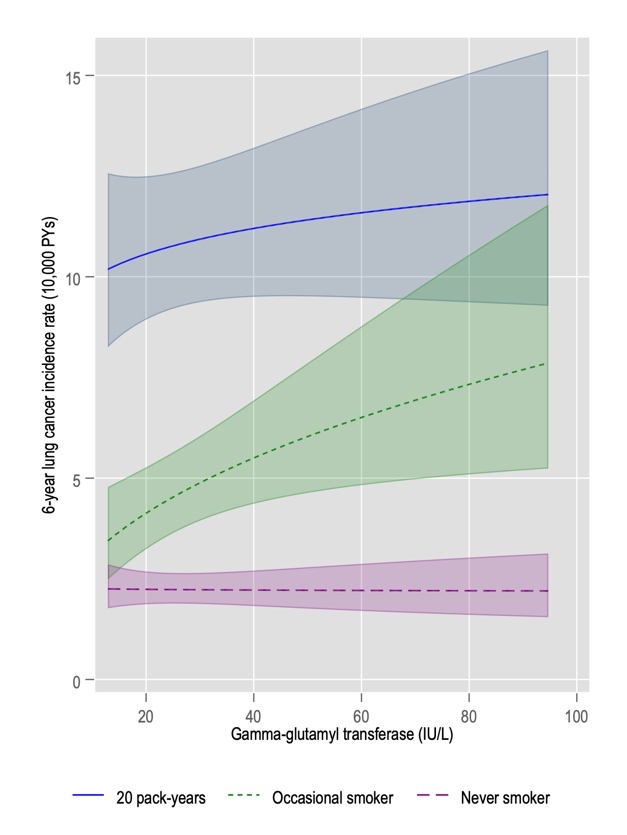


### Figure S2-d: Alkaline phosphatase and predicted lung cancer incidence


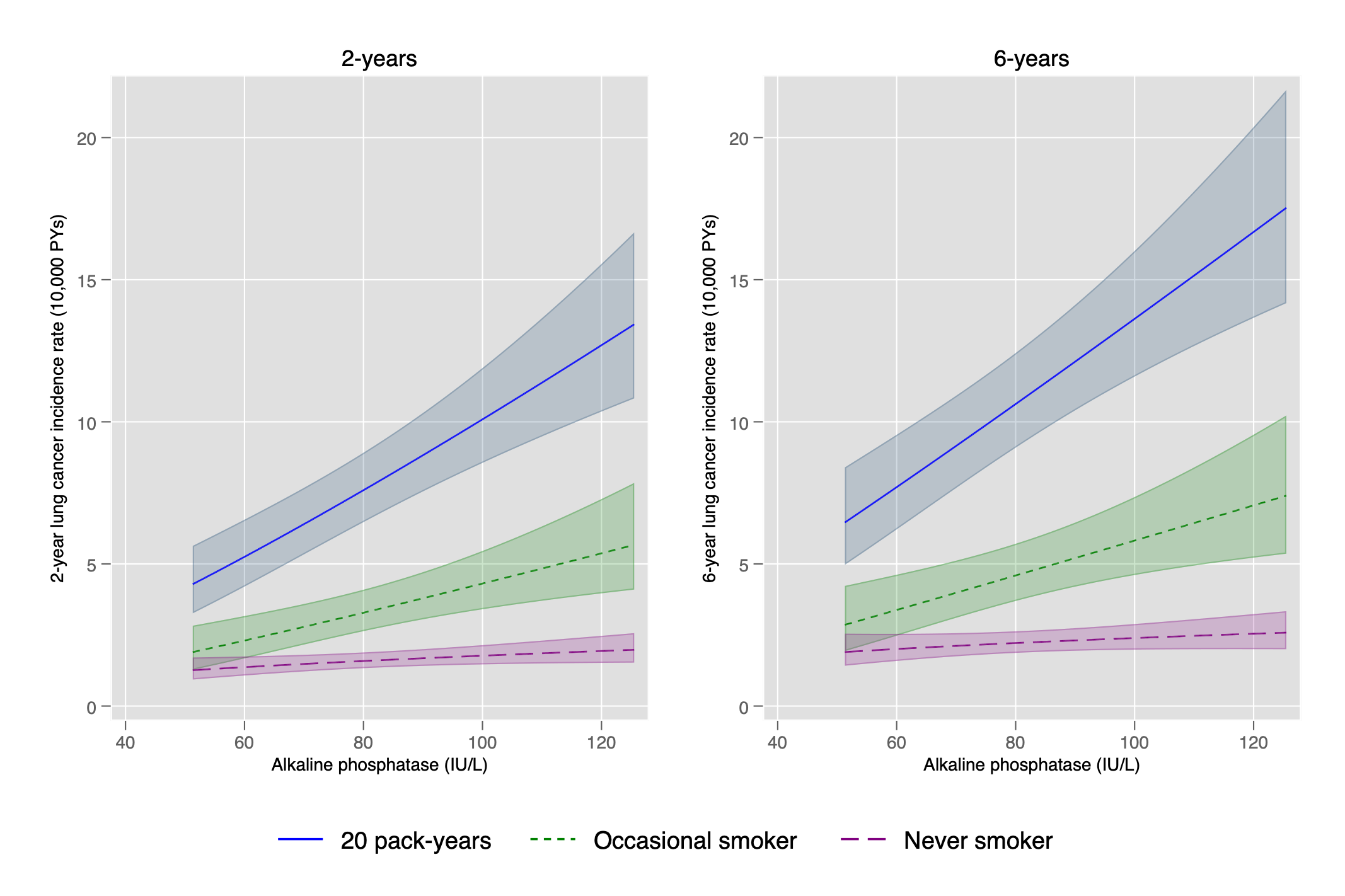


### Figure S2-e: Aspartate aminotransferase and predicted lung cancer incidence


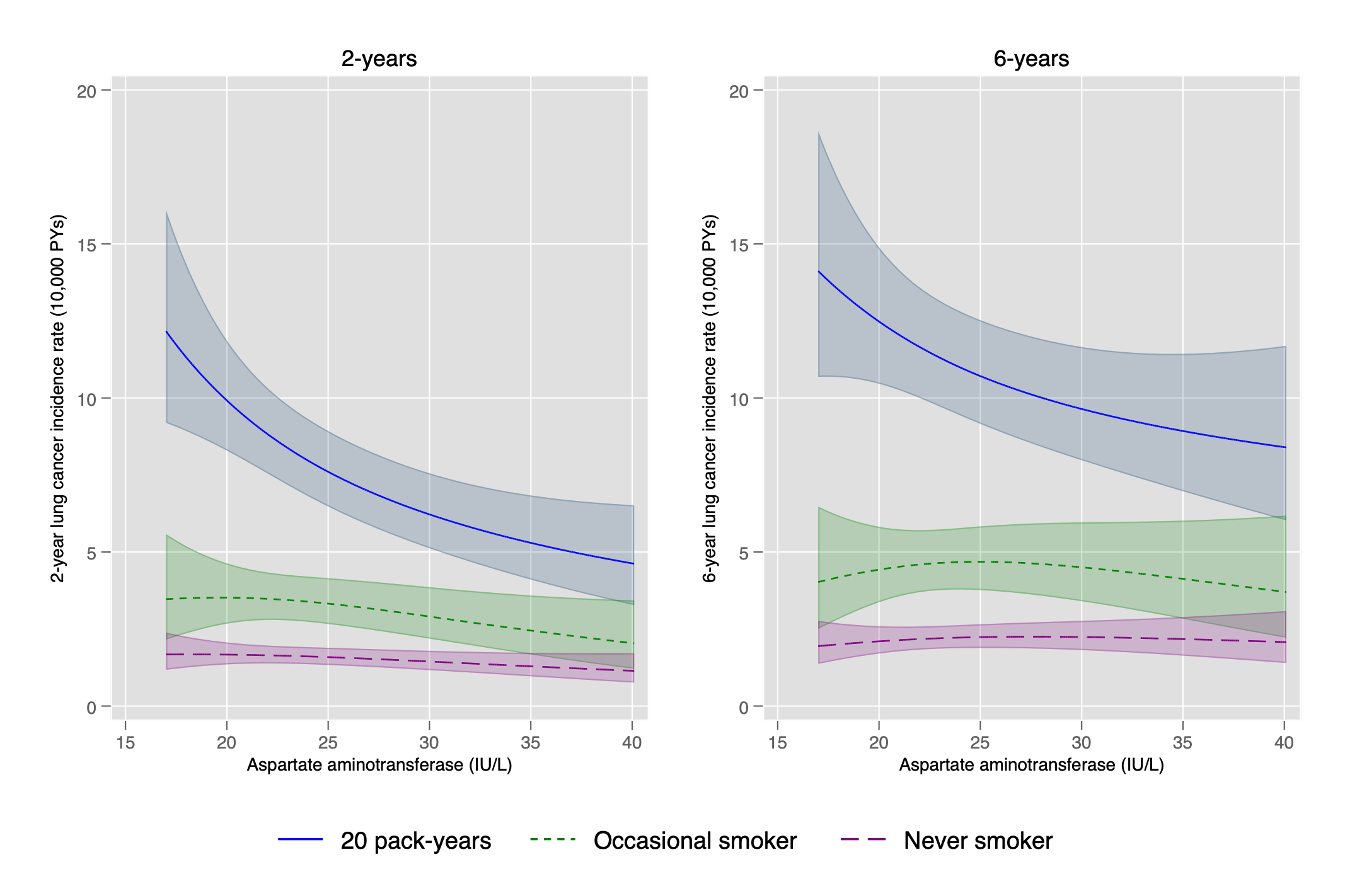


### Figure S2-f: Alanine aminotransferase and predicted lung cancer incidence


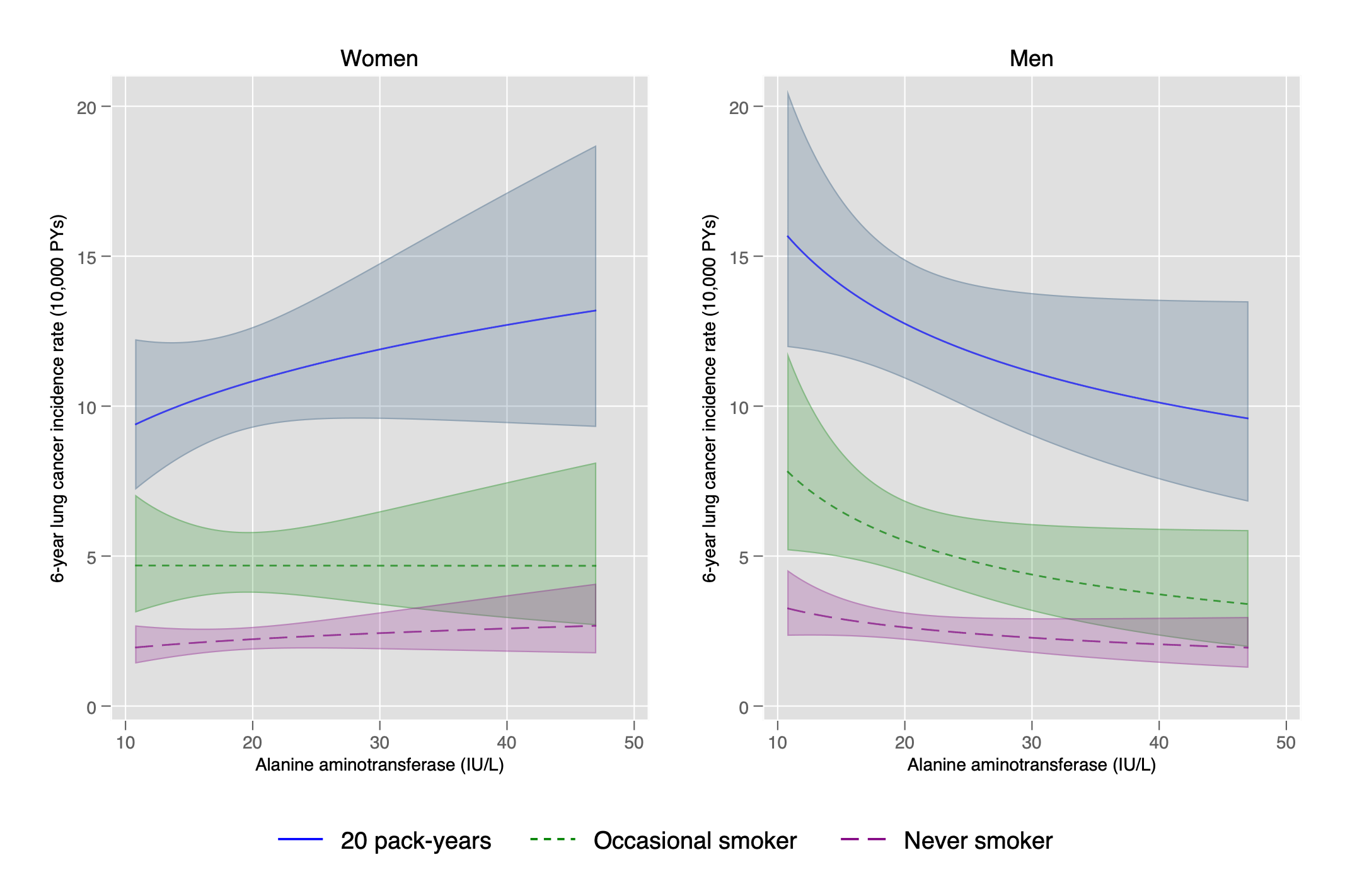


### Figure S2-g: Albumin and predicted lung cancer incidence


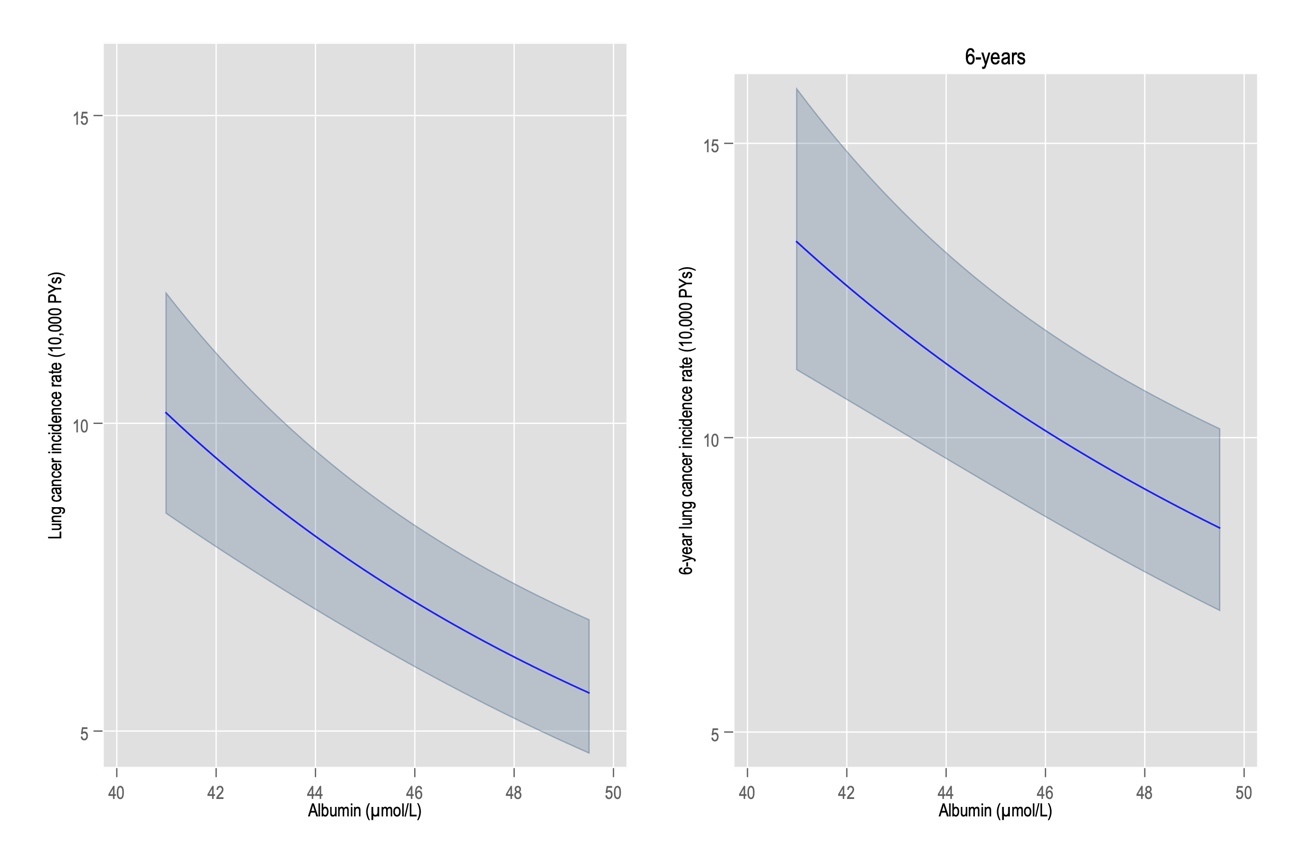


## Supplementary Tables

### Table S1: Blood-based measurements included in the panel

| Main use in primary health care | Biomarker | Sample Type | Supplier | Analysis method | Instrumentation |
| --- | --- | --- | --- | --- | --- |
| Liver function | Albumin | Serum | Beckman Coulter | AU5800 | BCG |
| Liver function | Total Bilirubin | Serum | Beckman Coulter | AU5800 | Photometric colour |
| Liver function | Gamma Glutamyltransferase | Serum | Beckman Coulter | AU5800 | IFCC |
| Liver function | Alanine aminotransferase | Serum | Beckman Coulter | AU5800 | IFCC |
| Liver function | Aspartate aminotransferase | Serum | Beckman Coulter | AU5800 | IFCC |
| Liver/Bone function | Alkaline Phosphatase | Serum | Beckman Coulter | AU5800 | AMP (IFCC) |
| Renal function | Urate Serum | Serum | Beckman Coulter | AU5800 | Uricase-PAP |

AMP= 2-Amino-2-Methyl-1-Propanol; BCG=Bromocresol Green; IFCC= International Federation of Clinical Chemistry

### Table S2: C-index and fraction of new information for different lung cancer risk models with functional forms and interactions selected using the Bayesian Information Criteria.

|  | All participants | C-index (95%CI) | Degrees of freedom | Model likelihood ratio test (χ²) | Likelihood ratio test p-value versus Scenario 1 | Fraction of new information* | Heuristic shrinkage factor** |
| --- | --- | --- | --- | --- | --- | --- | --- |
| Scenario 1 | Basic model | 0.805 (0.794 to 0.816) | 24 | 3613 |  |  | 0.99 |
| Scenario 2 | Basic model + FEV_1_ + alcohol + waist circumference | 0.811 (0.800 to 0.822) | 28 | 3777 | 2.47E-34 | 0.04 | 0.99 |
| Scenario 3 | Basic model + liver blood tests + urate | 0.809 (0.798 to 0.820) | 31 | 3722 | 1.78E-20 | 0.03 | 0.99 |
| Scenario 4 | Scenario 2 + 3 | 0.814 (0.803 to 0.825) | 35 | 3861 | 1.13E-46 | 0.06 | 0.99 |

FEV_1_=forced expiratory volume in 1 second

* Fraction of new information calculated as one minus the ratio of χ² value for Scenario 1 to the χ² value for the alternative scenario.

** Shrinkage factor = (χ² value - degrees of freedom)/χ² value

### Table S3: Changes in predicted risk of lung cancer across different risk models applied to 106,738 UK Biobank participants with a history of ever smoking and aged 55–74 years with functional forms and interactions selected using the Bayesian Information Criteria.

|  |  | C-index (95%CI) | Fraction of new information compared with Scenario 1* | Risk of lung cancer at 2 years | Risk of lung cancer at 6 years |
| --- | --- | --- | --- | --- | --- |
|  | |  |  | Median predicted risk % (IQR) | Median predicted risk % (IQR) |
| Scenario 1 | Basic model | 0.715 (0.700 to 0.729) | Ref | 0.124 (0.069 to 0.311) | 0.506 (0.281 to 1.268) |
| Scenario 2 | Basic model + FEV_1_ + alcohol + waist circumference | 0.731 (0.717 to 0.745) | 0.09 | 0.121 (0.065 to 0.299) | 0.497 (0.268 to 1.221) |
| Scenario 3 | Basic model + liver blood tests + urate | 0.726 (0.712 to 0.740) | 0.07 | 0.123 (0.067 to 0.303) | 0.501 (0.272 to 1.234) |
| Scenario 4 | Scenario 2 + 3 | 0.738 (0.725 to 0.752) | 0.14 | 0.120 (0.064 to 0.293) | 0.491 (0.260 to 1.196) |

FEV_1_=forced expiratory volume in 1 second

* Fraction of new information calculated as one minus the ratio of χ² value for Scenario 1 to the χ² value for the alternative scenario.

### Table S4: Characteristics at cohort recruitment and c-index in the training and validation sample following 1:1 split and using the Akaike Information Criterion for model specification.

|  | | Training sample | Validation sample |
| --- | --- | --- | --- |
|  | | N=250,920 | N=250,919 |
| Male | | 114,514 (45.6%) | 114,174 (45.5%) |
| Lung cancer during follow-up | | 1,320 (0.5%) | 1,323 (0.5%) |
| Age at cohort entry | | 58.3 (50.6-63.7) | 58.3 (50.6-63.7) |
| Weight | | 78.0 (15.5) | 78.0 (15.5) |
| Maximum height recorded | | 168.5 (9.2) | 168.5 (9.2) |
| Smoking status | | 136,940 (54.6%) | 136,434 (54.4%) |
| Never | | 86,362 (34.4%) | 86,262 (34.4%) |
| Former | | 26,202 (10.4%) | 26,696 (10.6%) |
| Current | | 1,416 (0.6%) | 1,527 (0.6%) |
| Screening scenario | | **C-index in validation sample (95%CI)** | **Fraction of new information compared with Scenario 1 in validation sample*** |
| Scenario 1 | Basic model (conventional risk predictors) | 0.818 (0.803 to 0.833) | Ref |
| Scenario 2 | Basic model + FEV_1_ + alcohol + waist circumference | 0.824 (0.809 to 0.838) | 0.046 |
| Scenario 3 | Basic model + liver blood tests + urate | 0.821 (0.806 to 0.836) | 0.040 |
| Scenario 4 | Scenario 2 + 3 | 0.823 (0.808 to 0.838) | 0.083 |
| Screening scenario restricted to ever smokers | |  |  |
| Scenario 1 | Basic model (conventional risk predictors) | 0.775 (0.757 to 0.793) | Ref |
| Scenario 2 | Basic model + FEV_1_ + alcohol + waist circumference | 0.788 (0.771 to 0.805) | 0.100 |
| Scenario 3 | Basic model + liver blood tests + urate | 0.783 (0.765 to 0.800) | 0.098 |
| Scenario 4 | Scenario 2 + 3 | 0.792 (0.776 to 0.809) | 0.176 |

FEV_1_=forced expiratory volume in 1 second

* Fraction of new information calculated as one minus the ratio of χ² value for Scenario 1 to the χ² value for the alternative scenario.

### Table S5: C-index following multiple imputation and using Bayesian Information Criterion for model specification.

| Screening scenario | | C-index (95%CI) | Median fraction of new information compared with Scenario 1 across n=10 imputed datasets (range)* |
| --- | --- | --- | --- |
| Scenario 1 | Basic model | 0.810 (0.801 to 0.820) | Ref |
| Scenario 2 | Basic model + FEV_1_ + alcohol + waist circumference | 0.821 (0.811 to 0.830) | 0.061 (0.054-0.065) |
| Scenario 3 | Basic model + liver blood tests + urate | 0.815 (0.806 to 0.825) | 0.038 (0.034-0.040) |
| Scenario 4 | Scenario 2 + 3 | 0.823 (0.814 to 0.832) | 0.085 (0.076-0.087) |
| Screening scenario restricted to ever smokers | |  |  |
| Scenario 1 | Basic model | 0.709 (0.697 to 0.721) | Ref |
| Scenario 2 | Basic model + FEV_1_ + alcohol + waist circumference | 0.736 (0.724 to 0.747) | 0.130 (0.084-0.176) |
| Scenario 3 | Basic model + liver blood tests + urate | 0.724 (0.712 to 0.736) | 0.138 (0.087-0.181) |
| Scenario 4 | Scenario 2 + 3 | 0.744 (0.732 to 0.755) | 0.122 (0.080-0.167) |

FEV_1_=forced expiratory volume in 1 second

* Fraction of new information calculated as one minus the ratio of χ² value for Scenario 1 to the χ² value for the alternative scenario.

### Table S6: Unit costs used in the economic model

| *Resource* | Cost inflated to 2022 values (£)* | Included in screening scenario | Reference |
| --- | --- | --- | --- |
| *Spirometry (non-hospital)* | 63.42 | 2 and 4 | [8] |
| *General practice case finding cost* | 20.57 | All scenarios | [8] |
| *Liver blood tests/urate* | 7.16 | 3 and 4 | [9] |
| *Computed tomography scan (one region)* | 92.77 | All scenarios | [10] |

*Costs inflated to 2022 values using the CCEMG – EPPI-Centre Cost Converter v.1.6: <https://eppi.ioe.ac.uk/costconversion/default.aspx> [11]

### Table S7: Cost per case detected of hypothetical lung cancer screening scenarios (using BIC for selecting interactions and non-linear transformations) applied to 106,738 UK Biobank participants with a history of ever smoking cigarettes.

| Screening Scenario | | Total cost of initial screening via telephone contact (n=106,738)* (£) | Per patient cost of follow-up tests to recalculate risk (£) | Total cost of follow-up tests after initial screening for those with a 1.51% risk over 6 years (n=23,333) (£) | No. meeting LDCT referral risk threshold (1.51% over 6 years) | Total cost LDCT screens for those meeting risk threshold (£)** | No. meeting LDCT referral risk threshold and with a lung cancer diagnosis by 6 years | Estimated cases detected at first screen*** | Cost per case detected (£) |
| --- | --- | --- | --- | --- | --- | --- | --- | --- | --- |
| 1 | Basic model | 2,195,601 | None | None | 23,333 | 2,195,601 | 807 | 161 | 27,015 |
| 2 | Basic model + FEV_1_ + alcohol + waist circumference | 2,195,601 | 63.42 | 2,195,601 | 19,590 | 3,675,380 | 753 | 151 | 36,472 |
| 3 | Basic model + liver blood tests + urate | 2,195,601 | 7.16 | 3,675,380 | 20,429 | 2,362,665 | 769 | 154 | 27,684 |
| 4 | Scenario 2 + 3 | 2,195,601 | 70.58 | 2,362,665 | 18,723 | 3,842,444 | 749 | 150 | 37,246 |

BIC=Bayesian Information Criterion; LDCT=Low-dose computed tomography; FEV_1_=forced expiratory volume in 1 second

*Cost of initial screen by health professional using telephone contact to be £20.57[8]. See supplementary information for further information on costs, resource use and assumptions on screening effectiveness.

**Cost of a low-dose computed tomography scan for one body region is £92.77 according to National Service Reference Costs for years 2019/21 and inflated to 2022 values[10].

***Estimated 20% of lung cancer diagnoses are detected at the first screen based on the NELSON European trial of LDCT screening[6].

### Table S8: Cost per case detected of hypothetical lung cancer screening scenarios (using AIC for selecting interactions and non-linear transformations in the training sample) applied to 53,467 UK Biobank participants in the validation sample with a history of ever smoking cigarettes.

| Screening Scenario | | Total cost of initial screening via telephone contact (n=53,467)* (£) | Per patient cost of follow-up tests to recalculate risk (£) | Total cost of follow-up tests after initial screening for those with a 1.51% risk over 6 years (n=12,173) (£) | No. meeting LDCT referral risk threshold (1.51% over 6 years) | Total cost LDCT screens for those meeting risk threshold (£)** | No. meeting LDCT referral risk threshold and with a lung cancer diagnosis by 6 years | Estimated cases detected at first screen*** | Cost per case detected (£) |
| --- | --- | --- | --- | --- | --- | --- | --- | --- | --- |
| 1 | Basic model | 1,099,816 | None | None | 12,173 | 1,099,816 | 302 | 60 | 36,906 |
| 2 | Basic model + FEV_1_ + alcohol + waist circumference | 1,099,816 | 63.42 | 1,099,816 | 7,934 | 1,871,828 | 249 | 50 | 52,367 |
| 3 | Basic model + liver blood tests + urate | 1,099,816 | 7.16 | 1,871,828 | 8,468 | 1,186,975 | 243 | 49 | 40,587 |
| 4 | Scenario 2 + 3 | 1,099,816 | 70.58 | 1,186,975 | 7,075 | 1,958,987 | 239 | 48 | 54,714 |

AIC=Akaike Information Criterion; LDCT=Computed tomography; FEV_1_=forced expiratory volume in 1 second

*Cost of initial screen by health professional using telephone contact to be £20.57[8]. See supplementary information for further information on costs, resource use and assumptions on screening effectiveness.

**Cost of a low-dose computed tomography scan for one body region is £92.77 according to NHS National Schedule of Reference Costs for years 2019/20 and inflated to 2022 values[10].

***Estimated 20% of lung cancer diagnoses are detected at the first screen based on the NELSON European trial of LDCT screening[6].

### Table S9: Cost per case detected of hypothetical lung cancer screening scenarios with varying risk thresholds for screening referrals applied to UK Biobank participants with a history of ever smoking cigarettes.

| Screening Scenario | | Townsend quintile (least deprived) | Townsend quintile 2 | Townsend quintile 3 | Townsend quintile 4 | Townsend quintile 5 (most deprived) |
| --- | --- | --- | --- | --- | --- | --- |
|  | **AIC for interactions and non-linear transformations (validation sample)** | **Cost per case detected (£)** | | | | |
| 1 | Basic model | 48,527 | 47,356 | 34,696 | 27,904 | 17,024 |
| 2 | Basic model + FEV_1_ + alcohol + waist circumference | 64,356 | 68,784 | 45,461 | 39,006 | 24,776 |
| 3 | Basic model + liver blood tests + urate | 50,467 | 49,687 | 35,121 | 28,554 | 17,284 |
| 4 | Scenario 2 + 3 | 64,158 | 68,646 | 45,592 | 38,413 | 25,646 |

AIC=Akaike Information Criterion; LDCT =Low-dose computed tomography; FEV_1_=forced expiratory volume in 1 second

### Table S10: Cost per case detected of hypothetical lung cancer screening scenarios (using AIC for selecting interactions and non-linear transformations in the training sample) applied to 53,467 UK Biobank participants in the validation sample with a history of ever smoking cigarettes.

|  | | Risk threshold for LDCT referral | | |  |
| --- | --- | --- | --- | --- | --- |
| Screening Scenario | | 0.05% over 6 years | 3% over 6 years | 5% over 6 years |  |
|  | **AIC for interactions and non-linear transformations** | **Cost per case detected (£)** | | | |
| 1 | Basic model | 31,000 | 33,420 | 69,492 |  |
| 2 | Basic model + FEV_1_ + alcohol + waist circumference | 44,671 | 42,952 | 87,707 |  |
| 3 | Basic model + liver blood tests + urate | 31,118 | 36,944 | 84,384 |  |
| 4 | Scenario 2 + 3 | 45,633 | 44,178 | 92,329 |  |

AIC=Akaike Information Criterion; LDCT =Low-dose computed tomography; FEV_1_=forced expiratory volume in 1 second

# Supplementary References

1. *Yujun Lian, 2014. "WINSOR2: Stata module to winsorize data," Statistical Software Components S457765, Boston College Department of Economics, revised 25 Nov 2020.*

2. Harrell, F.E., *Regression modeling strategies: with applications to linear models, logistic and ordinal regression, and survival analysis*. Vol. 3. 2015: Springer.

3. Royston, P. and P.C. Lambert, *Flexible parametric survival analysis using Stata: beyond the Cox model*. Vol. 347. 2011: Stata Press College Station, TX.

4. Harrell, F.E., Jr, et al., *Evaluating the Yield of Medical Tests.* JAMA, 1982. **247**(18): p. 2543-2546.

5. Harrell, F.E., Jr *Statistically Efficient Ways to Quantify Added Predictive Value of New Measurements. Last updated on 2020-11-15. Available from:* [*https://www.fharrell.com/post/addvalue/#disqus_thread*](https://www.fharrell.com/post/addvalue/#disqus_thread).

6. de Koning, H.J., et al., *Reduced Lung-Cancer Mortality with Volume CT Screening in a Randomized Trial.* N Engl J Med, 2020. **382**(6): p. 503-513.

7. Balata, H., et al., *Analysis of the baseline performance of five UK lung cancer screening programmes.* Lung Cancer, 2021. **161**: p. 136-140.

8. Lambe, T., et al., *Model-based evaluation of the long-term cost-effectiveness of systematic case-finding for COPD in primary care.* Thorax, 2019. **74**(8): p. 730-739.

9. *National Clinical Guideline Centre. Draft for consultation. Preoperative tests: Routine preoperative tests for elective surgery. Clinical guideline. Appendix M: Economic considerations for Delphi. October 2015.*

10. *Department of Health, NHS England, and NHS Improvement . Reference Cost Collection: National Schedule of Reference Costs, 2019–2020 - NHS trusts and NHS foundation trusts. London: NHS Improvement; 2020.*

11. Shemilt, I., T. James, and M. Marcello, *A web-based tool for adjusting costs to a specific target currency and price year.* Evidence & Policy, 2010. **6**(1): p. 51-59.
